# Improved electrical performance of a sol–gel IGZO transistor with high-k Al_2_O_3_ gate dielectric achieved by post annealing

**DOI:** 10.1186/s40580-019-0194-1

**Published:** 2019-07-22

**Authors:** Esther Lee, Tae Hyeon Kim, Seung Won Lee, Jee Hoon Kim, Jaeun Kim, Tae Gun Jeong, Ji-Hoon Ahn, Byungjin Cho

**Affiliations:** 1 Department of Advanced Material Engineering, Chungbuk National University, Chungbuk, 28644 Republic of Korea; 2 Department of Electronic Material Engineering, Korea Maritime and Ocean University, Busan, 49112 Republic of Korea

**Keywords:** Indium gallium zinc oxide IGZO, Post annealing, Capacitance–voltage measurement, X-ray photoelectron spectroscopy depth profiling, Electrical bias stress stability

## Abstract

We have explored the effect of post-annealing on the electrical properties of an indium gallium zinc oxide (IGZO) transistor with an Al_2_O_3_ bottom gate dielectric, formed by a sol–gel process. The post-annealed IGZO device demonstrated improved electrical performance in terms of threshold variation, on/off ratio, subthreshold swing, and mobility compared to the non-annealed reference device. Capacitance–voltage measurement confirmed that annealing can lead to enhanced capacitance properties due to reduced charge trapping. Depth profile analysis using X-ray photoelectron spectroscopy proved that percentage of both the oxygen vacancy (V_O_) and the hydroxyl groups (M–OH) within the IGZO/Al_2_O_3_ layers, which serve as a charge trapping source, can be substantially reduced by annealing the fabricated transistor device. Furthermore, the undesired degradation of the contact interface between source/drain electrode and the channel, which mainly concerns V_O_, can be largely prevented by post-annealing. Thus, the facile annealing process also improves the electrical bias stress stability. This simple post annealing approach provides a strategy for realising better performance and reliability of the solid sol–gel oxide transistor.

**Electronic supplementary material:**

The online version of this article (10.1186/s40580-019-0194-1) contains supplementary material, which is available to authorized users.

## Introduction

Recently, materials and process designs of transistor backplane circuits have emerged indispensable for next-generation display applications [1, 2]. Thin-film transistors are therefore a vital device to meet the requirements of practical optoelectronics. In particular, transistors using amorphous metal oxide semiconductors such as zinc tin oxide, indium zinc oxide, and indium gallium zinc oxide (IGZO) have attracted immense interest owing to their advantages of high optical transparency, high film uniformity in large-scale fabrication, and high field effect mobility [3–6]. Among the amorphous oxide semiconductors, IGZO is one of the most attractive materials owing to its excellent electrical and optical properties, stability, and low-temperature fabrication processability [7–10]. In particular, the high mobility of amorphous IGZO is attributed to the overlap of neighbouring indium metal orbitals and the oxygen vacancy (V_O_) acting as an electron donor [3]. In contrast, gallium prevents the formation of excess charge carriers and a leakage current in the off-state, while zinc makes the chemical structure of IGZO more stable. Even when a high-quality amorphous metal oxide is prepared from typical vacuum deposition techniques (such as magnetron sputtering, chemical vapor deposition, and atomic layer deposition) [9–11], the processes are relatively less competitive due to high fabrication costs and a long process time. Thus, solution processes that are low-cost, have large-area coating capability, and a high throughput rate can be a viable alternative [6, 12]. Nevertheless, the most serious drawback of solution-processed transistors is poor electrical performance. Recent studies have thereby focused on enhancing the performance of solution-processed transistor devices by optimising the composition [13, 14], modulating the carrier transport in the oxide semiconductor [15, 16], or introducing a high-k dielectric [17]. Most studies typically require heat treatment to complete the chemical reaction of the coated oxide thin films. In particular, the post-annealing step is very critical, as it can improve or, in some cases, degrade the electrical characteristics of the transistor due to a change in the chemical and physical properties of the channel, dielectric, and interface. Thus, it is highly critical to do the thorough study about the influence of the post-annealing on the transistor device.

In this work, the effects of post-annealing treatment on the electrical properties of IGZO transistors with an Al_2_O_3_ gate dielectric were investigated. The post-annealed transistor devices showed significantly improved performance in terms of the hysteresis window (ΔV_th_), mobility, and on/off ratio compared to the non-annealed reference device. The enhanced electrical properties were also confirmed by C–V measurement of the Al/Al_2_O_3_/p–Si capacitor structure. The enhanced performance was primarily correlated with relative atomic percentages of metal–oxygen (M–O), oxygen vacancy (V_O_), and hydroxyl bonding (M–OH). Depth profile analysis by X-ray photoelectron spectroscopy (XPS) showed that atomic percentages of V_O_ and M–OH, which degrade the transistor performance, were considerably reduced throughout the IGZO/high-k Al_2_O_3_ layers. Finally, it was also confirmed that the electrical instability arising from charge trapping defect states can be resolved by this simple post-annealing process.

## Materials and experimental methods

### Preparation of metal oxide precursor solutions

Precursor solutions (0.1 M) for the IGZO channel were prepared by mixing and dissolving the metal precursors (comprising indium nitrate hydrate, gallium nitrate hydrate, and zinc nitrate), in 2-methoxyethanol (2-ME). It is known that an increased indium molar ratio in an IGZO film leads to larger numbers of V_O_ and interstitial zinc, thereby creating more electrons [18]. Because gallium suppresses electron carriers, the gallium/zinc ratio is particularly important for controlling the electron carrier concentration [19]. Thus, an optimised ratio of each precursor is highly desirable. In our case, the mixed IGZO solution is composed of a 10:1:2 mass ratio of indium, gallium, and the zinc precursors respectively. For preparing the Al_2_O_3_ dielectric layer, an aluminium nitrate precursor (0.5 M) was dissolved in 2-ME. The solution was then stirred for over 12 h at 70 °C followed by filtration using a hydrophobic polytetrafluoroethylene (PTFE) syringe filter with a pore size of 0.2 μm.

### Fabrication of IGZO transistors

Firstly, p + Si substrates were sequentially cleaned with acetone, ethanol, and deionised water and then dried under nitrogen, followed by UV-ozone treatment for 10 min to remove impurities and to make the surface hydrophilic. Using the Al_2_O_3_ precursor solution prepared in the previous section, the dielectric film was spin-coated at 3000 rpm for 30 s under nitrogen. A two-step annealing process was performed next: the first step at 300 °C for 30 min and the second step at 500 °C for 30 min under air. After the UV-ozone treatment, the IGZO precursor solution was spin-coated onto the Al_2_O_3_/p + Si substrate. The coated IGZO films were then annealed at 350 °C for 1 h on a hotplate under air. The IGZO channel patterns were made by a conventional photolithography process, following which the IGZO film layer was subjected to wet etching. Finally, 50 nm-thick aluminium source and drain electrodes were deposited using a shadow mask with a thermal evaporator. The fabricated IGZO/Al_2_O_3_/p–Si transistor device next underwent post-annealing treatment at 200 °C for 1 h under air; for comparison, a non-annealed reference device was also fabricated.

### Characterisation of IGZO transistors

The elemental binding state of the oxide layers was analysed by XPS. Electrical characterisation consisted of measuring the current–voltage (I–V) and capacitance–voltage (C–V) properties using the Keithley 2636B and Keithley 4200-SCS with 4200-CVU modules, respectively.

## Results and discussion

**Fig. 1:**
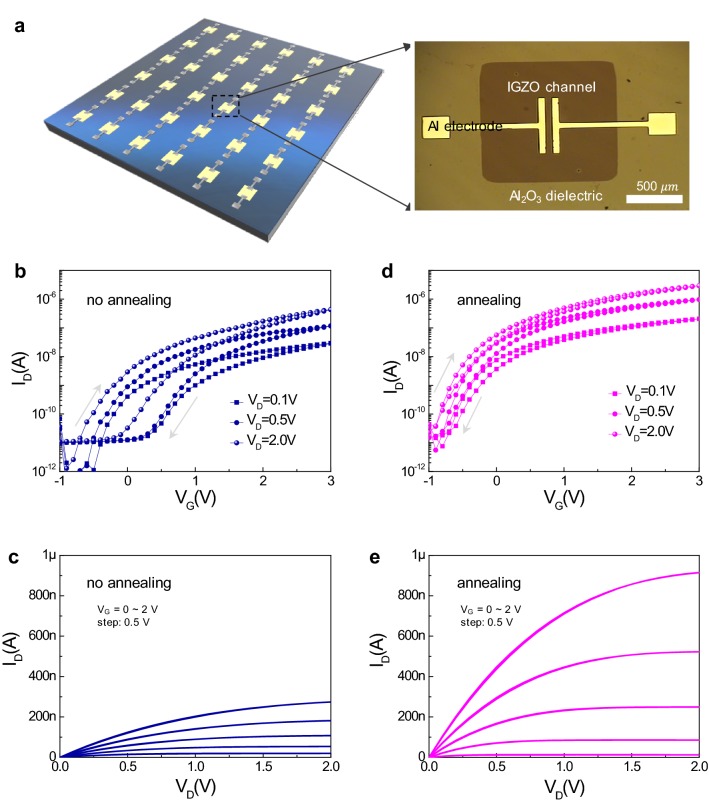
**a** Schematic of IGZO transistor array devices. The inset shows an optical microscope image of a single IGZO device with a channel region (50 μm length and 500 μm width). Transfer characteristics (drain current–gate voltage of I_D_–V_G_) of **b** reference IGZO transistor without post-annealing and **d** IGZO transistor with post-annealing at variable V_D_ (0.1, 0.5, and 2 V). Output characteristics (I_D_–V_D_) of **c** reference IGZO transistor and **e** IGZO transistor with post-annealing at variable V_G_ from 0 to 2 V

Figure 1a illustrates a schematic image of the IGZO transistor array devices. The inset presents an optical microscope image of a single IGZO device with a channel region 50 μm in length and 500 μm in width. The thickness of each layer was confirmed through the focused ion beam scanning electron microscopy image (Additional file 1: Figure S1). The thicknesses of aluminium electrode, Al_2_O_3_ dielectric layer, and IGZO channel layer were measured to be ~ 50, 10, and 50 nm, respectively. To check the effect of post-annealing on the IGZO transistor device, we compared the electrical characteristics of two kinds of transistor devices: the device with post-annealing versus the reference without post-annealing. Transfer characteristics, (i.e. drain current–gate voltage of I_D_–V_G_) of the reference were checked (Fig. 1b). The I_D_–V_G_ curves measured at various drain voltages (V_D_ of 0.1, 0.5, and 2 V) show large hysteresis curves with a clockwise direction. The on current of the transistor device was measured as ~ 4.89 × 10^−7^ A at V_D_ of 2 V. The on/off ratio was calculated as 6.39 × 10^3^ at V_D_ of 2 V. The hysteresis window may be attributed to charge trapping occurring from unintentional defect sources (e.g. impurities and vacancies) present in the active channel layer, dielectric, and interfaces. The underlying mechanism of the charge trapping effect will be discussed later in detail. Figure 1c shows the output characteristics (I_D_–V_D_) of the IGZO transistor device without post-annealing at a variable V_G_ ranging from 0 to 2 V. Overall, the reference device showed a relatively low output current. Conversely, the on current of the IGZO transistor with post-annealing was ~ 2.85 × 10^−6^ A, which was even higher than for the reference device (Fig. 1d). The on/off ratio was also increased by approximately ten times after annealing. The remarkable feature exhibited by the annealed device was that the large hysteresis phenomenon in the transfer curves had disappeared. As shown in Fig. 1e, the output characteristics are also much improved after post-annealing.

**Fig. 2:**
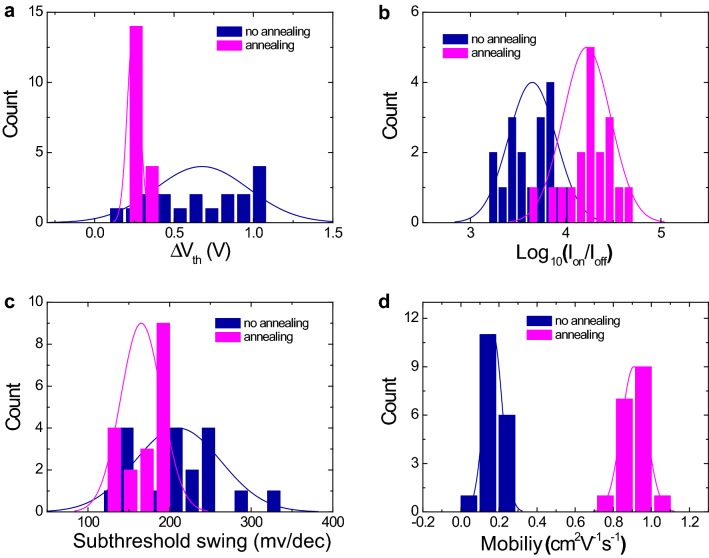
Histogram of **a** delta threshold voltage (∆V_th_: difference between two threshold voltage values obtained from double sweep curves), **b** on/off ratio, **c** subthreshold swing, and **d** mobility calculated at V_G_ sweeping rage of − 1 to 3 V at a fixed V_D_ of 0.1 V for no annealed IGZO transistor devices and annealed IGZO transistor devices

In order to check the statistical distribution of electrical parameters for the transistor array devices, we compared the electrical performance of each different IGZO transistor device (reference and control devices; 18 devices each). The histogram in Fig. 2a represents the delta threshold voltage (i.e. ∆V_th_, which is the difference between two threshold voltage values obtained from double sweep curves) for the reference IGZO devices that showed a wide range of 0.2–1 V, while the ∆V_th_ range of the control IGZO devices was very narrow at 0.2–0.3 V. The post-annealing process can thereby reduce diverse trap states within the IGZO transistor structure. The on current value was significantly improved after post-annealing of the fabricated final devices, whereas the off current values showed no noticeable change. Figure 2b shows that the on/off ratio was increased by about one order of magnitude. Figure 2c compares the subthreshold swing (SS) values between the annealed and non-annealed devices. Those devices with low SS values (i.e. a steep subthreshold slope) demonstrate a faster transition between off and on currents. Compared with the reference device, a relatively low and narrow SS value distribution was achieved for the annealed device, leading to a relatively fast switching property. A higher average field effect mobility value of ~ 0.97 cm^2^/V s was also found in the annealed devices compared to the reference of 0.17 cm^2^/V s (Fig. 2d). The statistical comparison among the essential transistor parameters indicates that the overall performance can be improved by this simple post-annealing process. The improved properties of the annealed device might be related to changes in the chemical state of IGZO and Al_2_O_3_ sol–gel films and to the interface properties of the Al/IGZO/Al_2_O_3_ stacked layers. Also, there was no significant change in the roughness of each layer before and after the post annealing. It suggests that the performance enhancement is more strongly related with the thermodynamical change in non-stoichiometry of In/Gallium/Zinc/Oxygen elements rather than any morphological change of the film roughness (Additional file 1: Figure S2). The average values of basic electrical parameters assessed for the two types of IGZO transistors were summarised and compared again (Additional file 1: Table S1). In addition, the electrical performances of the IGZO transistors were compared at different post annealing temperature. It turns out that its performance could be optimized at the post annealing temperature of 200 °C (Additional file 1: Figure S3). Post annealing effect on devices with different Al_2_O_3_ dielectric layer thickness were compared, showing the similar performance enhancement trend such as reduction in hysteresis window and increase in I_ON_ regardless of thickness of the dielectric layer (Additional file 1: Figure S4). In particular, our post annealing approach was so still effective even when other complex techniques for performance enhancement of IGZO based transistor were compared in terms of essential electrical parameters such as on/off ratio, SS value, and mobility (Additional file 1: Table S2).

**Fig. 3:**
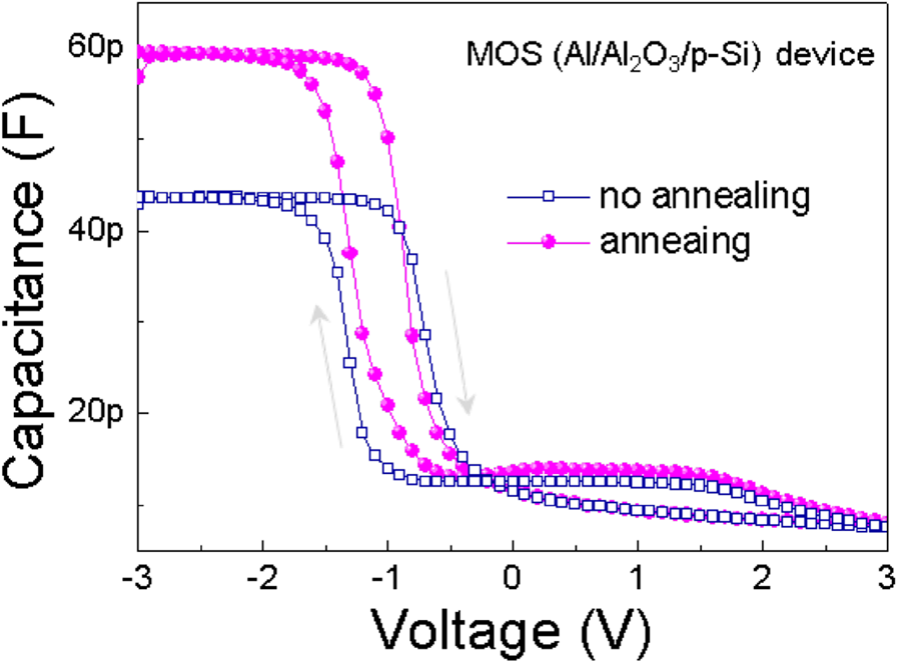
Comparison of C–V characteristics for non-annealed and annealed MOS capacitors at 1 MHz

In order to first check the effect of the post-annealing process on the dielectric property, we fabricated the Al/Al_2_O_3_/p–Si metal–insulator–semiconductor (MOS) capacitor structure, and then tested its C–V characteristic. The obtained C–V curves (Fig. 3) at a frequency of 1 MHz were compared between the two kinds of capacitors (i.e. the devices with or without post-annealing). When a negative voltage was applied to the Al electrode, major hole carriers accumulated at the surface of the p + Si substrate, leading to the formation of a charge dipole. Conversely, when a positive voltage was applied to the top electrode, hole carriers were withdrawn from the Al_2_O_3_/p–Si interface, causing a depletion region and thereby decreased capacitance. Interestingly, all the devices showed a clockwise direction to the hysteresis curve, indicating that a hole trapping process had occurred [20]. The hysteresis windows of the two capacitors were also very different, where the non-annealed and annealed capacitors provided respective values of 0.67 and 0.36. The suppression in C–V hysteresis is considered to have arisen from the improved interface quality [21]. Generally, the hysteresis in the C–V measurement at a high frequency (1 MHz) is induced by a charge trap near the interface [21]. These charge trap states in the Al_2_O_3_ layer and the interface defect states prevent hole carriers from accumulating near the surface of the p–Si substrate under a negative voltage [22, 23]. In addition, the capacitance of the annealed capacitor is higher than that of the non-annealed capacitor. It is likely for M–OH bonds and V_O_ to weaken polarisation in the Al_2_O_3_ dielectric [24]. The polarisation mechanism that predominantly occurs in Al_2_O_3_ dielectrics is based on the distortion of Al–O bonds, which breaks the original symmetry of lattice atoms under the applied electric field [25]. Thus, more M–OH bond and V_O_ reduce the number of distorted Al–O bonds contributing to the ionic polarisation, leading to decreased capacitance in the non-annealed reference capacitor.

**Fig. 4:**
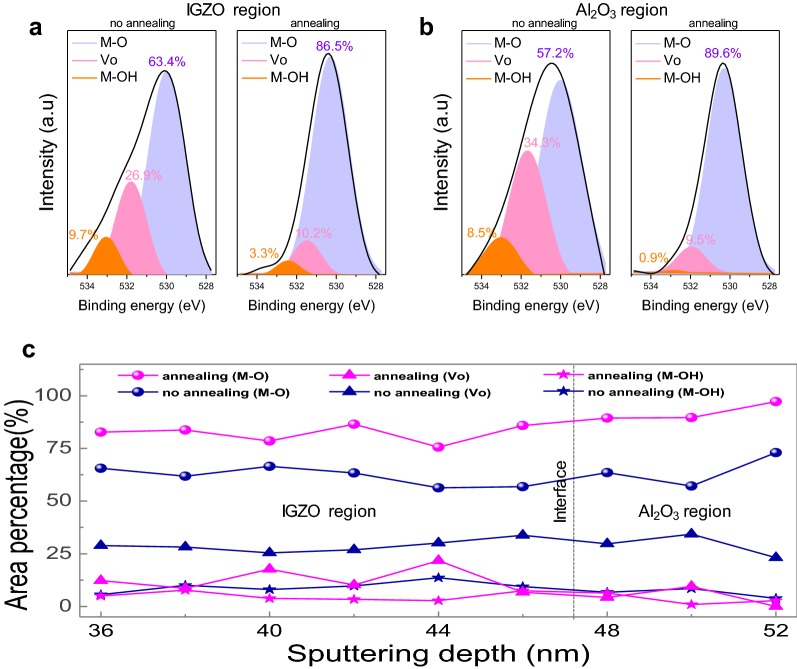
Deconvoluted XPS spectra (M–O, V_O_, and M–OH) of O 1S in **a** IGZO channel region and **b** Al_2_O_3_ dielectric region before and after post-annealing. **c** XPS depth profile representing atomic percentages of M–O, V_O_, and M–OH before and after post-annealing of IGZO/Al_2_O_3_ layers

The chemistry of the IGZO/Al_2_O_3_ layers was analysed by XPS on two samples: the IGZO/Al_2_O_3_/p–Si sample with and without post-annealing. In particular, oxygen binding energy states for the IGZO and Al_2_O_3_ regions were explored according to sputtering depth. The oxygen binding peaks could be deconvoluted to three peaks corresponding to M–O, V_O_, and M–OH binding. Figure 4a shows a significant change in the oxygen binding energy states in the IGZO region after annealing, where the percentage of M–O bonding has increased from 63.4 to 86.5%. Concomitantly, the V_O_ bonding decreases from 26.9 to 10.2% and likewise, the M–OH bond decreases from 9.7 to 3.3%. It is noteworthy that the annealing process could decrease the M–OH bonding states which prevent charge transport through metallic and oxygen orbital regions of the IGZO layer. Hydroxyl groups react with the surrounding H_2_ molecules during post-annealing, in a sequential process of M–OH decomposition, removal of volatile H_2_O as a by-product, and formation of M–O bonds [26]. In addition, oxygen ions diffuse and recombine with V_O_ via thermal annealing, thereby reducing V_O_ generated due to the non-stoichiometry of IGZO [27]. Figure 4b shows the change in the oxygen binding energy states in the Al_2_O_3_ layer before/after annealing; the percentage of M–O bonding increases from 57.2 to 89.6%, whereas that of V_O_ bonding decreases from 34.3 to 9.5% and that of the M–OH bond decreases from 8.5 to 0.9%. The considerable losses in the M–OH and V_O_ percentage exhibit almost the same trend as in the IGZO region. We needed to confirm whether such trends can be observed at different depths. Figure 4c shows the XPS depth profile representing the area percentage of M–O, V_O_, and M–OH binding energy states according to sputtering depth. Overall, a considerable decrease in M–OH and V_O_ binding is clearly observed for the IGZO/Al_2_O_3_ layers undergoing annealing. It has been reported that improved transistor performance after post-annealing is due to a reduced surface roughness of the IGZO active channel [28, 29]. Nonetheless, we found that a key factor for improving our transistor was the considerable suppression of M–OH and V_O_, as confirmed by the in-depth XPS results. It is generally known that M–OH bonds are more critical for inducing the charge trapping effect as opposed to V_O_ primarily as an electron donor [27]. We thus consider that the M–OH bonds can be a strong indicator for estimating the charge trapping density [30, 31].

**Fig. 5:**
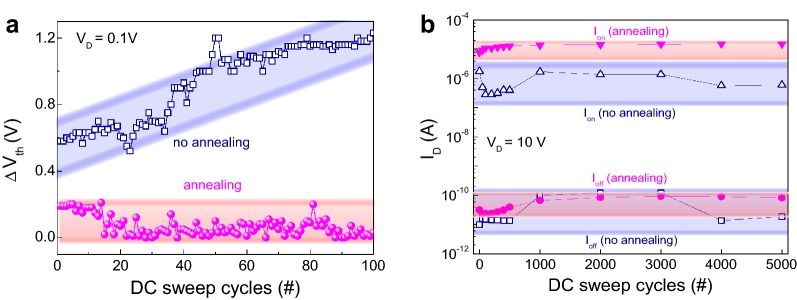
**a** Variation in threshold voltage shift during a 100 DC cycling test (V_D_ = 0.1 V) and **b** variation in the on and off current during a 5000 cycling test (@ V_D_ = 10 V) before and after post-annealing of the IGZO transistor

It should be noted that the chemical bond states M–O, V_O_, and M–OH, can also affect the bias stress stability. Figure 5a compares the changes in ΔV_th_ for the transistor devices (with and without post-annealing) during a repetitive 100 DC sweep cycling test under bias conditions of V_D_ (fixed voltage of 0.1 V) and V_G_ (sweep voltage of − 1 to 3 V). The variation in V_th_ for non-annealed IGZO transistor devices gradually increased from 0.7 to 1.2 V. However, in the annealed devices, the variation in V_th_ was small in itself (within 0.2 V), and likewise relatively small compared to the non-annealed transistor device, thereby indicating that the IGZO transistor has stable electrical properties. In order to check the bias stress stability of the physical interface between the channel and electrode, we also applied a relatively large drain voltage (V_D_ of 10 V and V_G_ of − 1 to 3 V) to the IGZO-based devices. Figure 5b shows the on and off data for the testing devices as function of the number of DC sweep cycles. The raw data of the linear transfer curves for each device are also displayed in Additional file 1: Figure S5. The on and off data for the post-annealed device remained stable without serious current fluctuation when compared to the non-annealed transistor. The wide variation in current (blue colour) of the non-annealed device during the cycling test indicates electrical degradation on the interface between the channel and electrode due to a large acceleration of electron carriers toward the interface. The reference device with larger V_O_ and M–OH bond percentages exhibits weakened stability due to the increased charge trapping sites [32, 33]. Indeed, electrical stability properties are more seriously dominated by interface defects as well as defects in the channel film. In particular, a large concentration of V_O_ at the interface induces an increased electrode metal reactivity, leading to a degradation in the electric performance and stability [34]. This unintentional reaction with V_O_ at the interface inducing electrical instability can thus be mitigated through a simple post-annealing process. The facile post-annealing process improves the electrical bias-stress stability, thereby extending the application to other oxide-based semiconductors. However, instead of the post annealing process to induce thermal degradation of polymer substrates, deep-ultraviolet annealing process will be alternatively applied for the flexible applications.

## Conclusions

In this study, we analysed the effects of a post-annealing treatment on the electrical performance and stability of IGZO transistors fabricated by a sol–gel process. The post-annealed transistor delivered superior electrical performance, in terms of a high on/off ratio, higher field effect mobility, and low SS values when compared to a non-annealed transistor. Reasons for this improvement were established by C–V characterisation and XPS depth analysis. The improved C–V properties (suppression in C–V hysteresis and a relatively high capacitance value) after post-annealing are strongly related to the reduced charge trap states and increased asymmetric Al–O bonds. XPS depth analysis proved that the substantial decrease in the atomic percentage of M–OH and V_O_ binding energy states, which play a main role as charge trap states, can lead to an improved annealing transistor performance. Furthermore, the unintentional defect reaction with V_O_ at the interface which induces electrical instability can be prevented by a straightforward post-annealing process. This approach may thus provide a simple way toward optimising the performance and improving the reliability of sol–gel oxide-based transistors.

## Additional file

**Additional file 1: Figure S1.** FIB SEM image of the IGZO-based FET device. **Figure S2.** AFM images of Al2O3 surface (a) before and (b) after post annealing. AFM images of IGZO surface (c) before and (d) after post annealing. **Figure S3.** (a) Transfer characteristics of IGZO transistor devices with different post annealing temperature (50 ~ 300 °C). Comparison of electrical parameters such as (b) Ion/Ioff, ratio, (c) ΔVth, and (d) mobility for devices treated with different post annealing temperature. **Figure S4.** Comparison of the transfer curves of the IGZO-based FET devices with different Al2O3 dielectric layer thickness (a) before and (b) after post annealing. **Figure S5.** Comparison of transfer curves during 5000 cycling test for (a) no annealing and (b) annealing device. **Table S1.** Comparison of electrical parameters between the IGZO transistor devices without post-annealing and the devices with post-annealing. **Table S2.** Comparison of processing techniques studied for performance improvement of IGZO/Al2O3 transistor devices.

## Data Availability

The authors have no data to share since all data are shown in the submitted manuscript.
