# Supplementary material for: Improved electrical performance of a sol–gel IGZO transistor with high-k Al2O3 gate dielectric achieved by post annealing
Source: Nano Converg. 2019 Jul 22;6:24. doi: 10.1186/s40580-019-0194-1 (PMC6643007; doi:10.1186/s40580-019-0194-1)
Supplement: Supplementary file 1 — Additional file 1: Figure S1. FIB SEM image of the IGZO-based FET device. Figure S2. AFM images of Al2O3 surface (a) before and (b) after post annealing. AFM images of IGZO surface (c) before and (d) after post annealing. Figure S3. (a) Transfer characteristics of IGZO transistor devices with different post annealing temperature (50 ~ 300 °C). Comparison of electrical parameters such as (b) Ion/Ioff, ratio, (c) ΔVth, and (d) mobility for devices treated with different post annealing temperature. Figure S4. Comparison of the transfer curves of the IGZO-based FET devices with different Al2O3 dielectric layer thickness (a) before and (b) after post annealing. Figure S5. Comparison of transfer curves during 5000 cycling test for (a) no annealing and (b) annealing device. Table S1. Comparison of electrical parameters between the IGZO transistor devices without post-annealing and the devices with post-annealing. Table S2. Comparison of processing techniques studied for performance improvement of IGZO/Al2O3 transistor devices. [file 40580_2019_194_MOESM1_ESM.docx]

Additional Information

**Improved electrical performance of a sol–gel IGZO transistor with high-k Al_2_O_3_ gate dielectric achieved by post annealing**

Esther Lee^1,†^, Tae Hyeon Kim^1,†^, Seung Won Lee^2^, Jee Hoon Kim^1^, Jaeun Kim^1^, Tae Gun Jeong^1^, Ji-Hoon Ahn^2^, and Byungjin Cho^1,*^

^1^Department of Advanced Material Engineering, Chungbuk National University,

Chungbuk 28644, Republic of Korea

^2^Department of Electronic Material Engineering, Korea Maritime and Ocean University, Busan 49112, Republic of Korea

*Correspondence: [bjcho@chungbuk.ac.kr](mailto:bjcho@chungbuk.ac.kr)

^†^Authors contributed equally to this work.

**Figure S1.** FIB SEM image of the IGZO-based FET device.

**Figure S2.** AFM images of Al_2_O_3_ surface (a) before and (b) after post annealing. AFM images of IGZO surface (c) before and (d) after post annealing.

**Figure S3.** (a) Transfer characteristics of IGZO transistor devices with different post annealing temperature (50 ~ 300 °C). Comparison of electrical parameters such as (b) I_on_/I_off_, ratio, (c) ΔV_th_, and (d) mobility for devices treated with different post annealing temperature.

**Figure S4.** Comparison of the transfer curves of the IGZO-based FET devices with different Al_2_O_3_ dielectric layer thickness (a) before and (b) after post annealing.


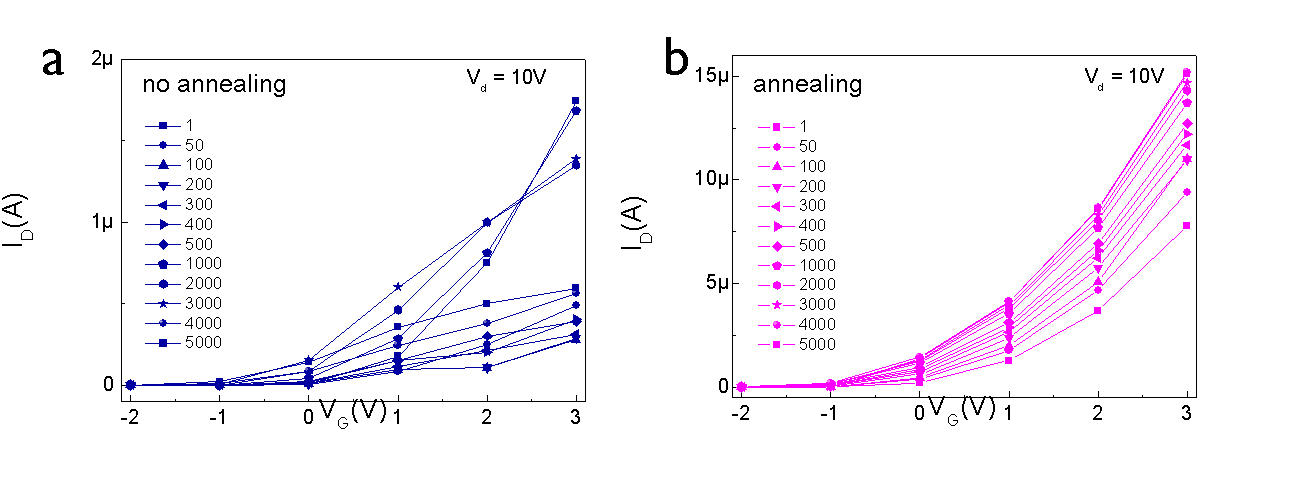


**Figure S5.** Comparison of transfer curves during 5000 cycling test for (a) no annealing and (b) annealing device.

**Table S1.** Comparison of electrical parameters between the IGZO transistor devices without post-annealing and the devices with post-annealing

| Type | V_D_ (V) | | I_on_ (A) | I_off_ (A) | I_on_/I_off_ | $\Delta$V_th_ (V) | S.S.  (mV/dec) | μ  (cm^2^V^-1^s^-1^) |
| --- | --- | --- | --- | --- | --- | --- | --- | --- |
| No annealing | | 0.1 | 3.87$\times$10^-8^ | 1.09$\times$10^-11^ | 3.52$\times$10^3^ | 0.70 | 206 | 0.16 |
|  |  | 2 | 4.89$\times$10^-7^ | 7.65E-10^-11^ | 6.39$\times$10^3^ | 0.44 | 214 | 0.13 |
| Post  annealing | | 0.1 | 1.9$\times$10^-7^ | 1.42$\times$10^-11^ | 1.33$\times$10^4^ | 0.25 | 165 | 0.90 |
|  |  | 2 | 2.85$\times$10^-6^ | 1.17$\times$10^-10^ | 2.42$\times$10^4^ | 0.21 | 174 | 0.81 |

**Table S2.** Comparison of processing techniques studied for performance improvement of IGZO/Al_2_O_3_ transistor devices

| processing technique | Electrical Parameter | before | after | Ratio (%)  $\frac{after-before}{before}\times100$ | Ref. |
| --- | --- | --- | --- | --- | --- |
| Post annealing | on/off | 3.52E+03 | 1.33E+04 | 278 | This work |
|  | SS | 206 | 165 | -20 |  |
|  | mobility | 0.16 | 0.9 | 462 |  |
| Al_2_O_3_ passivated  by thermal ALD | on/off | 9.59E+06 | 3.67E+04 | -100 | [S1] |
|  | SS | 250 | 330 | 32 |  |
|  | mobility | 8.6 | 10.1 | 17 |  |
| Al_2_O_3_ passivated  by remote plasma ALD | on/off | 9.59E+06 | 4.77E+06 | -0.5 | [S1] |
|  | SS | 250 | 210 | -16 |  |
|  | mobility | 8.6 | 7.8 | 9 |  |
| HfO_2_ buffer layer | on/off | 4.77E+06 | 1.32E+07 | 177 | [S1] |
|  | SS | 210 | 230 | 10 |  |
|  | mobility | 7.8 | 8 | 3 |  |

References

[S1] Y. B. Ko, S. W. Bang, S. J. Lee, S.Y. Park, The effects of a HfO2 buffer layer on Al2O3-passivated indium-gallium-zinc-oxide thin film transistors. Phys. Status solidi. 10-11. 403405 (2011)
